# Supplementary material for: Accurately Identifying Cerebroarterial Stenosis from Angiography Reports Using Natural Language Processing Approaches
Source: Diagnostics (Basel). 2022 Aug 3;12(8):1882. doi: 10.3390/diagnostics12081882 (PMC9406429; doi:10.3390/diagnostics12081882)
Supplement: Supplementary file 1 [file diagnostics-12-01882-s001.zip › Supplementary Table.pdf]

Supplementary Table S1. Examples of the keyword matching guideline for the rule-based NLP model.

Location and target		
● LACA: Left anterior cerebral artery; LIICA: Left internal carotid artery; LIVA: Left intracranial vertebral artery; LMCA: Left middle cerebral artery; and LPCA: Left posterior cerebral artery<br>● RACA: Right anterior cerebral artery; RIICA: Right internal carotid artery; RIVA: Right intracranial vertebral artery; RMCA: Right middle cerebral artery; and RPCA: Right posterior cerebral artery<br>● BA: Basilar artery		
	Keywords	Example sentences
Inclusion	>=50% stenosis/narrowing	● Significant stenosis at right MCA M1 segment and right distal VA.<br>● Faint hyperintensity at the pons without restricted water diffusion suggesting old infarction.<br>● Arteriosclerosis with tight stenosis of right proximal VA orifice.
	>50% stenosis	
	significant stenosis/narrowing	
	tight stenosis/narrowing	
	severe stenosis/narrowing	
	high grade stenosis/narrowing	
	high-grade stenosis/narrowing	
	high degree stenosis/narrowing	
	high-degree stenosis/narrowing	
	prominent stenosis/narrowing	
	critical stenosis/narrowing	
	(no/non/faint/poor) + (flow/visual/opacification/opacity)	
	Absence	
Exclusion	(not/no/without) + stenosis	● Nonsignificant stenosis at bilateral ICA.<br>● Focal stenosis at right V4 segment.<br>● Normal caliber of right carotid without stenosis.
	<50% stenosis	
	(segmental/focal/moderate) + stenosis	

Supplementary Table S2. Specificity results for stenosis detection by different models and results are expressed as mean ± standard deviation.

	Internal testing dataset (n = 1,922)			External testing dataset (n = 315)		
	Rule-based model	***LSTM***	***XLNet***	***Rule-based model***	***LSTM***	***XLNet***
RIICA	0.99 ± 0.00	0.99 ± 0.01	0.99 ± 0.00	1.00	1.00 ± 0.00	0.96 ± 0.08
RACA	1.00 ± 0.00	0.99 ± 0.00	1.00 ± 0.00	1.00	1.00 ± 0.00	1.00 ± 0.00
RMCA	0.99 ± 0.00	0.98 ± 0.00	0.99 ± 0.00	1.00	0.99 ± 0.01	0.99 ± 0.01
RPCA	1.00 ± 0.00	0.99 ± 0.00	1.00 ± 0.00	1.00	1.00 ± 0.00	1.00 ± 0.00
RIVA	0.99 ± 0.00	0.99 ± 0.01	0.99 ± 0.00	1.00	0.95 ± 0.07	0.98 ± 0.04
BA	0.99 ± 0.00	0.99 ± 0.00	0.99 ± 0.00	0.99	0.63 ± 0.21	0.90 ± 0.14
LIICA	0.99 ± 0.00	0.99 ± 0.01	0.99 ± 0.00	1.00	0.97 ± 0.07	0.89 ± 0.17
LACA	1.00 ± 0.00	0.99 ± 0.00	1.00 ± 0.00	1.00	1.00 ± 0.00	1.00 ± 0.00
LMCA	0.99 ± 0.00	0.98 ± 0.01	0.99 ± 0.00	1.00	0.98 ± 0.03	0.98 ± 0.01
LPCA	1.00 ± 0.00	0.99 ± 0.00	0.99 ± 0.00	1.00	1.00 ± 0.00	1.00 ± 0.00
LIVA	0.99 ± 0.00	0.99 ± 0.00	0.99 ± 0.00	1.00	0.94 ± 0.08	0.97 ± 0.06

Supplementary Table S3. Sensitivity results for stenosis detection by three different models and results are expressed as mean ± standard deviation.

	Internal testing dataset (*n* = 1,922)			External testing dataset (*n* = 315)		
	Rule-based model	***LSTM***	***XLNet***	***Rule-based model***	***LSTM***	***XLNet***
RIICA	0.86 ± 0.01	0.69 ± 0.06	0.85 ± 0.04	0.42	0.05 ± 0.08	0.52 ± 0.19
RACA	0.89 ± 0.03	0.45 ± 0.13	0.88 ± 0.05	0.00	0.00 ± 0.00	0.20 ± 0.33
RMCA	0.88 ± 0.02	0.61 ± 0.10	0.89 ± 0.02	0.15	0.10 ± 0.10	0.52 ± 0.14
RPCA	0.89 ± 0.02	0.52 ± 0.14	0.86 ± 0.02	0.00	0.00 ± 0.00	0.13 ± 0.12
RIVA	0.92 ± 0.01	0.85 ± 0.03	0.88 ± 0.02	0.50	0.05 ± 0.15	0.45 ± 0.35
BA	0.84 ± 0.03	0.68 ± 0.06	0.86 ± 0.04	0.67	0.22 ± 0.16	0.66 ± 0.10
LIICA	0.87 ± 0.03	0.75 ± 0.03	0.87 ± 0.04	0.50	0.32 ± 0.20	0.60 ± 0.25
LACA	0.90 ± 0.04	0.40 ± 0.17	0.89 ± 0.03	0.50	0.00 ± 0.00	0.57 ± 0.16
LMCA	0.88 ± 0.02	0.70 ± 0.08	0.88 ± 0.03	0.00	0.20 ± 0.14	0.72 ± 0.16
LPCA	0.86 ± 0.02	0.55 ± 0.12	0.86 ± 0.04	0.00	0.00 ± 0.00	0.00 ± 0.00
LIVA	0.91 ± 0.02	0.83 ± 0.04	0.87 ± 0.02	0.00	0.25 ± 0.25	0.45 ± 0.27

Supplementary Table S4. The area under the receiver operating characteristic curve results for XLNet model with different training epochs and learning rate in stenosis detection task and results are expressed as mean ± standard deviation.

	Internal testing dataset (n = 1,922)		External testing dataset (n = 315)	
	5 epochs with 1e-5 learning rate	***20 epochs with 2e-5 learning rate***	***5 epochs with 1e-5 learning rate***	***20 epochs with 2e-5 learning rate***
RIICA	0.98 ± 0.01	0.98 ± 0.01	0.93 ± 0.08	0.92 ± 0.08
RACA	0.98 ± 0.01	0.98 ± 0.01	0.88 ± 0.13	0.90 ± 0.11
RMCA	0.98 ± 0.01	0.98 ± 0.01	0.94 ± 0.07	0.92 ± 0.06
RPCA	0.97 ± 0.02	0.97 ± 0.01	0.84 ± 0.13	0.84 ± 0.07
RIVA	0.98 ± 0.01	0.98 ± 0.00	0.97 ± 0.08	0.91 ± 0.12
BA	0.97 ± 0.02	0.97 ± 0.01	0.81 ± 0.06	0.80 ± 0.10
LIICA	0.98 ± 0.01	0.98 ± 0.01	0.93 ± 0.09	0.88 ± 0.14
LACA	0.98 ± 0.02	0.97 ± 0.02	0.94 ± 0.07	0.96 ± 0.04
LMCA	0.98 ± 0.01	0.98 ± 0.00	0.96 ± 0.04	0.96 ± 0.07
LPCA	0.97 ± 0.01	0.97 ± 0.01	0.82 ± 0.12	0.64 ± 0.15
LIVA	0.97 ± 0.02	0.98 ± 0.01	0.93 ± 0.10	0.84 ± 0.14
